# Expert international trauma clinicians’ views on the definition, composition and delivery of reintegration interventions for complex PTSD

**DOI:** 10.1080/20008066.2023.2165024

**Published:** 2023-01-17

**Authors:** Maria Condon, Michael A. P. Bloomfield, Helen Nicholls, Jo Billings

**Affiliations:** a Division of Psychiatry, University College London, London, UK; b Translational Psychiatry Research Group, Mental Health Neuroscience Department, Division of Psychiatry, Institute of Mental Health, University College London, London, UK; c Traumatic Stress Clinic, St Pancras Hospital, Camden and Islington NHS Foundation Trust, London, UK; d National Hospital for Neurology and Neurosurgery, University College London Hospitals NHS Foundation Trust, London, UK; e National Institute for Health Research University College London Hospitals Biomedical Research Centre, London, UK

**Keywords:** CPTSD, treatment, reintegration, expert opinion, thematic analysis, TEPT-C, tratamiento, reintegración, opinión experta, Análisis temático, CPTSD, 治疗, 重新整合, 专业意见, 专题分析

## Abstract

**Background:** Research has previously distinguished between complex post-traumatic stress disorder (CPTSD) and PTSD, with the former including a range of disturbances in self-regulatory capacities in addition to difficulties associated with PTSD. Clinical guidelines have previously recommended a phase-based approach for the treatment of CPTSD, yet the final ‘reintegration’ phase of treatment has been overlooked in research, with limited evidence into its value and effectiveness, and inconsistencies in its definitions and understanding.

**Objective:** We set out to define and determine the key principles of ‘reintegration’ and to specify the components and method of delivery of treatment.

**Method:** Leading national and international clinical and academic experts in CPTSD were interviewed and asked about their views of how ‘reintegration’ should be defined, its role in the treatment of CPTSD, what it should be composed of, the key principles of its delivery, and how it should be evaluated. We analysed transcripts of the interviews following the principles of Codebook Thematic Analysis.

**Results:** We conducted 16 interviews with leading national and international experts with at least 10 years’ experience of treating people with CPTSD. Themes derived from our analysis demonstrated that while the definition and composition of reintegration varied greatly between experts, the key principles in its delivery were consistent across all experts.

**Conclusions:** The results of this study lay the foundation for a framework of what reintegration is and how it can be used in, but also highlight the need for more research to be conducted on the role of reintegration in the treatment of CPTSD. Consensus for the definition and composition of reintegration is still yet to be reached. Possible measures for evaluating reintegration should also be explored in the future.

## HIGHLIGHTS

- Reintegration is one of the recommended phases of treatment for CPTSD, however recent research has found that there are inconsistencies in its definition, role and value.
- The current study shows the opinions of experts, highlighting areas of consensus as well as differences in views with regards to the definition, composition and key principles in the delivery of reintegration.

## Abstract (es)

**Antecedentes:** La investigación ha distinguido previamente entre el trastorno de estrés postraumático complejo (TEPT-C) y el TEPT, y el primero incluye una variedad de trastornos en las capacidades de autorregulación además de las dificultades asociadas con el TEPT. Las pautas clínicas han recomendado previamente un enfoque basado en fases para el tratamiento del TEPT-C, pero la fase final del tratamiento de ‘reintegración’ se ha pasado por alto en la investigación, con evidencia limitada sobre su valor y efectividad, e inconsistencias en sus definiciones y comprensión.

**Objetivo:** Nos propusimos definir y determinar los principios clave de la ‘reintegración’ y especificar los componentes y el método de entrega del tratamiento.

**Método:** Se entrevistó a destacados expertos clínicos y académicos nacionales e internacionales en TEPT-C y se les preguntó sobre sus puntos de vista sobre cómo debería definirse la ‘reintegración’, su papel en el tratamiento del TEPT-C, de qué debería estar compuesta, los principios clave de su ejecución, y cómo se debe evaluar. Analizamos las transcripciones de las entrevistas siguiendo los principios del análisis temático de libro de códigos.

**Resultados:** Realizamos 16 entrevistas con destacados expertos nacionales e internacionales con al menos 10 años de experiencia en el tratamiento de personas con TEPT-C. Los temas derivados de nuestro análisis demostraron que, si bien la definición y la composición de la reintegración variaban mucho entre los expertos, los principios clave en su ejecución eran consistentes entre todos los expertos.

**Conclusiones:** Los resultados de este estudio sientan las bases para un marco de qué es la reintegración y cómo se puede utilizar, pero también resaltan la necesidad de realizar más investigaciones sobre el papel de la reintegración en el tratamiento del TEPT-C. Todavía no se ha llegado a un consenso sobre la definición y composición de la reintegración. Las posibles medidas para evaluar la reintegración también deberían explorarse en el futuro.

## Abstract (zh)

背景：先前的研究已经区分了复杂性创伤后应激障碍 (CPTSD) 和 PTSD，前者除了与 PTSD 相关的困难外，还包括一系列自我调节能力障碍。 临床指南此前曾建议采用阶段性方法治疗 CPTSD，但研究中忽视了治疗的最终‘重新整合’阶段，其价值和有效性的证据有限，其定义和理解也不一致。

目的：我们着手定义和确定‘重新整合’的关键原则，并指定其组成和实施治疗的方法。

方法：采访了 CPTSD 方面的国内和国际领先临床和学术专家，询问他们对‘重新整合’的定义、其在 CPTSD 治疗中的作用、它应该由什么组成、其实施的关键原则、 以及应该如何评估。 我们按照主题分析编码本的原则分析了访谈记录。

结果：我们对具有至少 10 年治疗 CPTSD 患者经验的国内和国际领先专家进行了 16 次采访。 从我们的分析中得出的主题表明，虽然重新整合的定义和组成在专家之间差异很大，但其实施的关键原则在所有专家中都是一致的。

结论：本研究的结果为重新整合是什么以及如何应用它的框架奠定了基础，但也强调需要对重新整合在 CPTSD 治疗中的作用进行更多研究。 关于重新整合的定义和构成的共识尚未达成。 今后还应探索评估重新整合的可能措施。

## 1. Introduction

The concept of complex post-traumatic stress disorder (CPTSD) was initially proposed by Herman (1992) to describe a syndrome experienced by survivors of repeated, prolonged or multiple traumas. However, only recently has CPTSD been formally introduced as an official diagnosis distinguishable from the diagnosis of PTSD in the eleventh revision of the International Classification of Diseases (ICD-11) (Worldwide Health Organisation, 2018). According to the ICD-11, a diagnosis of PTSD requires evidence of three ‘core’ symptoms, which include: re-experiencing the trauma, deliberate avoidance, elevated arousal and hypervigilance (Maercker et al., 2013). CPTSD requires the same criteria along with disturbances in self-organisation symptoms, including affect dysregulation, impairments in self-concept and disturbances in relationships (Brewin, 2019).

In its third revision, the International Society for Traumatic Stress Studies (ISTSS) guidelines on CPTSD noted the previous inconsistency in defining CPTSD, which has consequently led to issues with delivering treatment (Berliner et al., 2019). Mainly, the ISTSS has established that treatment for CPTSD is likely to involve more diverse interventions as well as longer duration of treatment, in comparison to PTSD. Therefore, not distinguishing between the two disorders may have led to less effective treatment options for those with CPTSD. Furthermore, research has consistently demonstrated that CPTSD, in comparison to PTSD, is associated with lower levels of functioning, increases in comorbidity and significant decreases in quality of life, suggesting that CPTSD is likely to be clinically more severe and also likely to require more intense treatment than for PTSD (Brewin et al., 2017; Cloitre et al., 2019; Karatzias & Cloitre, 2019). Karatzias et al. (2016) found that patients who were referred to trauma specialist centres more frequently presented with symptom profiles fitting the CPTSD criteria as opposed to PTSD, highlighting the need for evidence-based and acceptable treatment for CPTSD. Karatzias, Hyland, et al. (2019) further argue that these differences in complexity and severity of CPTSD should be expressed in individualised treatment that is specific to the diagnosis to ensure the efficiency of the intervention.

A phase-based approach for treating CPTSD was first suggested by Herman in 1992. Subsequently, the ISTSS proposed a phase-based protocol for treating CPTSD (Cloitre et al., 2012). The protocol consists of three distinct phases, each with a specific function; stabilisation, trauma memory processing and reintegration (Cloitre et al., 2012). The first stage, stabilisation, aims at ensuring the individual's safety, managing life stressors, increasing emotional and social skills as well as providing psychoeducation regarding trauma and CPTSD. The second stage, trauma memory processing, aims at reviewing and addressing traumatic memories that are accompanied by negative emotions, cognitions and beliefs, with the goal of processing traumatic memories and updating distressing cognitions and affect (Courtois & Ford, 2012).

Initial evidence for the use of a phase-based approach to treat CPTSD derived from a survey, finding that the majority of expert clinicians voted for a ‘sequenced’ phase-based approach as opposed to other treatment options (Cloitre et al., 2011). However, in contrast to this, De Jongh et al. (2016) argued against the superiority of a phased approach, emphasising the lack of direct evidence showing the benefits of this approach vs. trauma-focused psychotherapy without stabilisation, highlighting that previous literature has found that immediate trauma-focused treatment without stabilisation can provide necessary and sufficient support. De Jongh et al. advocate that the stabilisation phase is not necessary and may lead to delays in providing trauma-focused interventions. In support of this, a recent systematic review and meta-analysis (Karatzias, Murphy, et al., 2019) found that standard treatments for PTSD are successful in decreasing symptoms of negative self-concept and impairments in relationships for CPTSD, which brings into question the need for a phase-based approach. However, the studies and reviews presented by Karatzias et al. focused either on phase one, stabilisation, or phase two, trauma memory processing, neglecting any research on phase three.

The final stage, reintegration, has been inconsistently defined, with less clinical and research attention in comparison to the other two phases (McFetridge et al., 2017). Herman initially described reintegration as being ready to incorporate the lessons of the traumatic experience into the individual's life, with an emphasis on regaining power of control and trust (1992). Similarly, Cloitre et al. (2012) suggest that reintegration focuses on the transition out of therapy into community life. According to these authors, reintegration involves the therapist supporting the individual in applying skills to build safe relationships and social networks as well as to plan for the future. Additionally, reintegration has been argued to involve both identity and self-esteem development (Courtois, 2010). Finally, reintegration may also be a particularly challenging phase for individuals, as it requires realising the dysfunctional past, while continuing to move beyond its impact (Courtois & Ford, 2009).

Alongside inconsistency with the definition for the reintegration phase, there is also a lack of evidence about the composition of reintegration interventions. A recent systematic review of the effectiveness of reintegration interventions for CPTSD emphasised a striking absence of research, with most studies either completely overlooking the third phase or suggesting only weak evidence for its effectiveness (Purnell et al., 2021). As pointed out by Purnell et al. there is so far no existing evidence that systematically evaluates reintegration interventions, which is likely due to the previously mentioned issues with the reintegration phase being ill-defined and inconsistently conceptualised. Purnell and colleagues’ review identified only 15 studies published to date describing reintegration, which covered a range of interventions such as yoga, physical exercise, social activities, self-defense, and education. Other interventions described focused on relationships, trust and intimacy levels. The variety of outcome measures used across the existing research to examine reintegration interventions made it difficult to evaluate and compare their effectiveness.

A further important question in conceptualising reintegration also relates to defining the key principles of delivery: (1) Who should deliver reintegration? (2) How long should it last? (3) At what point in treatment should it be delivered? Previous research has provided no consensus in answer to these questions, with limited evidence only suggesting that it can be time consuming and should last between six and 12 months (Cloitre et al., 2011; McFetridge et al., 2017).

The updated ISTSS guidelines (2019) do not explicitly advocate for sequential phase-based treatment, but rather adopt a more flexible approach, highlighting the importance of individualised treatment. Nonetheless, the guidelines do not mention any treatment that focuses on reintegration interventions, while mainly discussing the importance of trauma-focused interventions.

Therefore, to address the inconsistency in definition and the lack of consensus for the composition and key principles of reintegration, this study draws on the findings from the systematic review by Purnell et al. (2021), aiming to define and determine the key principles of ‘reintegration’ and specify the components and method of delivery of treatment. Whilst the official diagnosis of CPTSD is relatively new and research on it still in its infancy, experienced clinicians have nevertheless long been treating people presenting with this constellation of difficulties and have long been providing ‘reintegration’ focused interventions based on Herman’s (1992) model, even in the absence of established consensus and evidence. We, therefore, sought to explore expert clinical opinion on reintegration amongst national and international experts in CPTSD, and particularly their views about its definition, composition and delivery.

## 2. Methods

Ethical approval was provided by University College London (Ref. 19937/001).

### 2.1. Participants and procedure

Participants were leading national and international clinical experts in CPTSD and leading clinical academics who had published extensively on the subject of CPTSD. Inclusion criteria were that the participant was a clinical or deputy clinical lead in a national specialist trauma service; and/or held a senior position in a national or international trauma organisation; and/or a leading clinical academic publishing extensively on CPTSD; and had at least 10 years’ experience of working clinically with people with CPTSD.

Participants were contacted through the contacts of JB and other eminent trauma specialists, through reviewing key research publications on CPTSD where the lead author was also a clinician involved directly in treatment of people with CPTSD, and through the following organisations: UKPTS, ESTSS, EJPT, ISTSS. We deliberately sought a range of participants who were the most established clinicians in the field of CPTSD. We purposively sought to include participants from a variety of settings, including different clinical work settings, client groups with CPTSD and geographical locations. All participants gave written informed consent to take part in the study. Interviews were conducted remotely online by MC. Participants provided socio-demographic information including place of work and country, professional role, client group worked with, length of time working with CPTSD clients, gender, age range and ethnic group.

### 2.2. Interview schedule

The semi-structured interview consisted of 11 open-ended questions, relating to the definition, practical clinical use, composition, key principles, and the evaluation of reintegration (see supplementary material). The questions were constructed based on prior literature on CPTSD, reintegration and phased-based interventions for treating CPTSD and through discussion with the research team as well as with an expert reference group of leading UK trauma clinicians.

### 2.3. Data analysis

The interviews were transcribed verbatim with all identifiable features of the participants removed. Codebook thematic analysis was used to analyse the data (Braun & Clarke, 2019). This approach was chosen as each interview consisted of an identical set of questions for each participant, which resulted in varied responses to the same questions. The transcripts were imported into NVivo and coded according to the initial research questions, with more inductive themes being identified which captured the experts’ responses. This approach allowed us to capture where there was consensus among experts along with exceptions and disagreements. The coding of the interview data was constantly discussed and revised between MC and JB.

## 3. Results

Sixteen leading experts were recruited for participation in study. The gender, roles, ethnicity, settings, geographical locations and years of work experience of the participants are shown in Table 1.

**Table 1.** Participant characteristics

Gender	
Female	9
Male	7
Role	
Clinical psychologist	4
Academic clinical psychologist	10
Psychiatrist	1
Counselling psychologist	1
Ethnicity	
White	13
(White British)	(7)
(White European)	(1)
(White Other)	(5)
Mixed Other	1
Mixed White	1
Asian	1
Setting^a^	
Public health service	10
University	10
Private practice	3
Charity	2
Geographical location	
UK	
England	10
Scotland	1
Wales	1
Switzerland	1
United States	1
Chile^b^	1
South Africa	1

^a^ Several participants worked across more than one setting, e.g. both at university and a public health service.

^b^ One participant located in South America had more than 5 but less than 10 years’ experience in treating CPTSD, due to this being a novel diagnosis there.

Interviews took place between 30th May and 14th December, 2021 and lasted between 23 and 52 min.

The main five themes were established deductively based on the research questions and the questions used in the interviews. The themes included: (1) Definition; (2) The value of reintegration; (3) Composition; (4) Key principles; (5) Evaluation. Within these themes, several sub-themes were identified more inductively from the experts’ responses, which are described below and illustrated with quotations (Table 2).

1. Definition

**Table 2.** Themes.

1. Definition of reintegration
1.1 Identity
1.2 Transferring skills from therapy to life
1.3 Future-oriented thoughts
1.4 Accessing resources
1.5 Enhancing life
1.6 Social strategies
2. The value of reintegration
2.1 What reintegration adds to treatment of CPTSD
3. Composition of reintegration
3.1 Functioning
3.2 Social integration
3.3 Physical well-being and exercise
3.4 Occupation
3.5 Emotion regulation
3.6 Symbolic rituals
3.7 Group work
4. Key principles of reintegration
4.1 Person-centred approach
4.2 Collaborative care
4.3 Realistic goals
4.4 Who reintegration is delivered by
4.5 When reintegration should be delivered
4.6 How long reintegration should last
5. Evaluation of reintegration
5.1 What is evaluated
5.2 How it is evaluated

This category describes how experts defined the phrase ‘reintegration’. There was significant variation in answers to this question, with many experts acknowledging challenges in defining reintegration and using terms to substitute for it, including ‘reclaiming’ or ‘rebuilding’.

1.1 Identity

The most commonly discussed theme when defining reintegration related to the concept of individual identity. Self-esteem, empowerment, managing interpersonal circumstances and helping the client to understand their own needs all contributed to a sense of self and to an understanding of their role within society.

People need a little bit more work around that to kind of even understand themselves and find themselves and see who they are or who could they be … 

A few experts talked about the importance of changing the individual's perception of self after working through the trauma, and learning to appreciate themselves as an individual:

… they can sort of reclaim life and move forward with a different view of themselves.

The focus on the identity of the individual was linked to many other concepts, but since reintegration, as defined by experts, seemed to revolve around helping the individual understand a sense of self, this was separated as a distinct theme.

1.2 Transferring skills from therapy to real life

Most experts talked about reintegration in terms of incorporating the skills learned in therapy into real life, ensuring that the individual is prepared for typical day-to-day tasks and interactions. This concept also related to enhancing life, discussed further in 1.5.

… it's one of those phases where the things that they have learnt in phase one and the work that we have done in phase two is sort of put into practice in a wider life.

1.3 Future-oriented

Thinking about the future, whether that is moving on from the trauma or setting goals, was another theme all experts mentioned while defining reintegration. Many referred to reintegration as the ‘moving forward phase’:

In an empowered way sort of moving forward, living in the present and seeing a future.

Some experts highlighted that reintegration also involves incorporating goals and building plans that are important and meaningful to the client.

1.4 Accessing resources

Many emphasised that the resources of the client should be assessed by the therapist at the start of treatment to identify the next steps of the reintegration process. These recourses may need to be developed to continue with reintegration interventions or, alternatively, reintegration would involve helping the individual understand how to use them and what is available to them:

… people will come into treatment with different resources, whether it is individual resources, so what's available in terms of their systems, so it is families, communities, as well as, you know, their geographical variation in terms of what's just available and where they are with things, and what do they want to achieve.

1.5 Enhancing life

A theme that was discussed by most experts when defining reintegration was that it ultimately involves enhancing the client's life. This could be about finding meaningful activities, creating new possibilities for people or improving daily functioning. This was particularly important, since many experts believed that the problem with the term ‘reintegration’ is that it implies that the person was previously a part of certain activities, a community or a life that they were happy with, which was not always the case.

… not just reclaiming, but claiming, sort of rebuilding or building, or achieving things that were never achieved or doing the things that they never did when they were growing up.

One international expert stressed the importance of not just finding meaningful activities, but also generally connecting with the world.

… help the patient get connected to the world and fulfil their duties, and find meaning in life, and, you know, more overarching goals.

The theme of bringing meaning to the individual's life seemed to be one of the key commonalities in defining reintegration between all experts.

1.6 Social strategies

One expert expressed that reintegration is also specifically about social strategies that ensure that the individual is functioning within the community. This would also relate to a sense of identity and functioning, but the emphasis on reintegration being about social guidance led this to be a separate theme.

… reintegration is social strategies, not health strategies, social strategies and social type of interventions to help someone return back to community, to become part of their community group … 

2. The value of reintegration

This theme describes experts’ views of the role of reintegration within the package of treatment for CPTSD and how it fits alongside the other ‘phases’ of treatment, as well as what it adds to treatment.

2.1 What reintegration adds to treatment of CPTSD

All experts perceived reintegration as an essential part of treatment, adding invaluable transferrable life skills that can be used outside of therapy. Most experts reflected on reintegration specifically being empowering to the person, while giving meaning to the entire course of therapy. This theme further expands on the importance of reintegration being about transferring skills to real life, as discussed in 1.2.

The reintegration phase is absolutely essential because if you do not assist the client or patient in fully translating what they gained and learned into their daily life and their relationships, and their sense of self on a day-to-day basis, then the treatment has really all just been talk or has all been just in the office or the therapeutic setting … but the extension out into the world is really the ultimate goal of treatment.

A few experts also highlighted that reintegration specifically was beneficial in preventing relapse, ensuring that the individual does not require mental health services anymore. One expert admitted that whilst reintegration might not help with symptom reduction it would, however, extend the benefits of the therapy:

The aim of the treatment is to overcome the symptoms of CPTSD. I don't think that reintegration strategies will help you with that, but I think that the reintegration strategies would help maintain the benefits of the treatment in the long run.

In contrast, another expert believed that reintegration, along with stabilisation and trauma-focused work, were all essential in treating the symptoms of CPTSD, and that neglecting reintegration could result in no change in symptom reduction:

I think they go hand-in-hand because you can take away these sorts of florid symptoms but you’re not touching all the awful and intractable impairment that's gone on in someone's lives. Actually, they’re not going to take any enjoyment in life, and that's going to lead to, you know, the dissociative, disorganisation symptoms in PTSD, you know, not changing at all.

3. Composition

Views about the composition of reintegration varied significantly across experts. The consensus amongst everyone, however, was that interventions should be bespoke to the individual.

3.1 Functioning

Almost all experts mentioned interventions that were intended to improve the functioning and life of the client. This section mainly consisted of examples of non-specific activities that, nonetheless, were consistently mentioned by the majority of experts.

I think just any kind of activities that would enhance their functioning and build their life outside of the CPTSD symptoms.

As mentioned in 1.5, experts also emphasised the importance of incorporating meaningful activities to enhance functioning, which would vary based on the individual.

3.2 Social integration

Social integration was highlighted by all experts. This could include creating new friendships or intimate relationships, as well as socialising within the community or building/rebuilding relationships.

A major issue with people with complex PTSD is loneliness, lack of activity … so if you pick up this quite early and … help them become part of their community.

Suggested interventions mainly focused on learning to trust again as well as developing social skills. Furthermore, a few experts stated that, where possible and if the client is willing, they would invite the client's close contacts to be a part of the treatment as well. This could include ‘safe peers’, family, partners, and friends.

3.3 Physical well-being and exercise

Approximately half of the experts mentioned physical well-being and exercise as a possible reintegration intervention. One expert stated that physical exercise is a key aspect of reintegration and should be recommended for everyone in one way or another.

… it just enhances the patient's, you know, sense of being a strong person. Physical exercise is something I talk to everybody about, all of my patients, you know, in some way or another.

Another international expert suggested using personalised physical exercise for people who were physically constrained, while acknowledging the individual's choices.

3.4 Occupation

Interventions relating to occupation were a common theme across all interviews. This could include finding new work, returning to education, enrolling in new courses or charity work – based on the client's preferences.

I would say the most typical example would be to enable someone to go back to education.

All the occupational interventions were argued as important in contributing towards sense of identity, well-being and sense of community.

And on the top of the list is returning to work … it is so important for a person's, you know, overall well-being and sense of being a useful person, useful member of the society.

3.5 Emotion regulation

Emotion regulation interventions were not overly common among responses but were still mentioned by a few experts as being important during reintegration. Firstly, emotional regulation interventions were argued to be an important skill to transfer to day-to-day life when feeling distressed or triggered. However, importantly, during the reintegration phase of treatment, these interventions were described more as a collaborative process, rather than teaching of a skill:

So, emotion regulation, again, if it's understood as not so much of a teaching of a skill, even though there can be some of that, but it's much more of accessing of capacities that the client has.

3.6 Symbolic rituals

Some participants described a variety of symbolic rituals, such as creating scrapbooks with ‘good days’, that they incorporated in their therapy during the reintegration phase. These could include anything important to the client, symbolising letting go of the trauma and moving on.

It can help the patient to sort of make that step on a symbolic level, where you, for instance, somehow burn or destroy some elements that remind you of the trauma … And the second part, second element of that ritual, would be something like, you know, celebrating re-establishing contact with the current world.

3.7 Group work

A few experts identified group settings as a helpful tactic in reintegration interventions. One expert described group reintegration interventions as useful in creating a feeling of connection between people, therefore assisting with social interactions as well. Another expert shared that group work may be under-estimated and could be more useful than anticipated:

… there is a real value in people connecting together who have had similar but different journeys, but like could help with those interpersonal skills that might be needed before you then fly out into the world.

4. Key principles

While there was a lot of diversity in defining reintegration, most experts followed the same principles for delivery, with most themes within this domain overlapping across all interviews.

4.1 Person-centred approach

All experts highlighted that utilising a person-centred approach was one of the key principles in delivering reintegration. This was based on the client's values, needs and their experiences. Importantly, many people added that what may work for one individual, may not work for another.

… an approach or intervention that is suitable for one person may well not be appropriate for another person.

4.2 Collaborative care

Participants described an important aspect of reintegration as listening to the client's values and encouraging them to make choices about their life, while being guided, when necessary, by the therapist. Therefore, a collaborative process seemed to be one of the main principles in successfully implementing reintegration.

One expert told us:

The clients choices need to be honoured as the basis for how you talk about what's happening in the client's life, what they choose to talk about, how they view it – that doesn't mean that the therapist can't provide perspectives and can't potentially help a client to make some shifts in how they’re thinking or in what they consider the most of crucial priorities or the best approach to handling situations or solving dilemmas.

Whilst experts acknowledged the value of the individual making choices about their life for themselves, some experts also recognised that this autonomy could bring up feelings of anxiety and other difficulties for the clients.

4.3 Realistic goals

Opinions differed when talking about setting goals for clients. While most believed that a key principle should be setting realistic goals to ensure that the person does not feel overwhelmed or like they will fail, another expert believed that through relentless optimism and constant goal-setting more success is achievable:

… setting ridiculous targets that are unachievable now to convey the message this is achievable, you can have a normal life, even if you’re going to have PTSD.

4.4 Who reintegration is delivered by

Responses to this question varied. Most experts agreed that the treating clinician should either start or lead the reintegration treatment. However, in addition to the therapist, many other services were mentioned in providing reintegration interventions: social care workers, charities, occupational therapists, marriage and family therapists, counsellors, psychiatrists, case managers, and peer support workers. Most experts also agreed that the most successful and beneficial treatment would be delivered by a multi-disciplinary team.

… the entire treatment team should deliver the reintegration component, and who that team is comprised of will vary for different clients.

However, some other experts argued that perhaps reintegration should not fall on the trauma therapist at all, suggesting that other services would be more suitable for providing reintegration:

I haven't thought that it should be delivered by experts in the field but the people who normally deliver those interventions in my setting and other settings are not necessarily clinicians. They’re people who have been involved in social care and more widely.

In contrast to this, another expert insisted that reintegration should solely be provided by the psychologist leading the case. However, an important aspect of this opinion was based on the availability of resources within the mental health system.

Finally, some experts reflected on the additional support people outside of therapy can provide for the client, such as family, friends, and partners, further reflecting the above-mentioned importance of social integration interventions (see 3.2).

4.5 When reintegration should be delivered

Most participants believed that reintegration work should start at the very beginning of therapy, albeit with perhaps a stronger emphasis on reintegration closer to the end of treatment. The reasoning behind this was that it helps build confidence and adds value to the therapy process.

… we start that already, you know, at the start of therapy, helping build people's confidence to start doing things.

Only one participant discussed reintegration being delivered solely at the end of treatment, arguing that some people are too vulnerable for reintegration work at the start.

The difference in opinions could reflect the idea of ‘accessing resources’ (see 1.4), suggesting that some people need to already have resources in place for reintegration interventions to begin.

4.6 How long reintegration should last

All participants struggled to answer how long reintegration should last. Most were opposed to the idea of placing a time point on the treatment, since it is so person-centred. Additionally, experts believed that reintegration was interweaved with the other ‘phases’, therefore naming a time scale for reintegration separately was difficult.

I have to say that that's not answerable except on an individual basis.

However, the most common response for a time scale was a minimum of 3 months. Others also stated a range from 3 months to several years, depending on the complexity of the trauma. Experts also gave a range of treatment sessions between 20 and 30 sessions, but this was relating to the entire treatment, rather than reintegration.

Importantly, some experts mentioned the benefit of having follow up/booster sessions with clients after they have left the therapy:

I would suggest that they would just have an opportunity to have some follow-ups, if they need to or the ability to maybe come back and work on some very specific things.

Only one expert working in privately with a specific cohort of people was able to dedicate solely the final session to reintegration, lasting approximately a few hours.

5. Evaluation

Answers relating to how reintegration should be evaluated involved two components: what was evaluated and how was it evaluated. According to the experts, there were as yet no clearly established ways of measuring reintegration.

5.1 What is evaluated

Participants discussed measuring quality of life, well-being, global functioning, social adjustment scales, satisfaction, and goal-based outcome measures. These measures are reminiscent of the focus on enhancing the individual's life through improving daily functioning, when defining reintegration (see 1.5). The most common response, although without any consensus on a specifically recommended measure, was to use a goal attainment measure.

I think there's kind of something about … measuring the individual goals and achievements of those goals, and satisfaction.

Some other experts suggested evaluating reintegration by measuring symptom reduction in contrast to measures relating to functioning. One expert argued that it would make sense to measure CPTSD symptoms as the predominant focus of the treatment.

5.2 How it is evaluated

This theme included discussion of clinician-based measures or subjective client-based assessments or feedback. Most experts recommended using a combination of both, combining objective and subjective measures.

… the best way to evaluate it is a combination of standardized functioning measures and personalized idiosyncratic goals … 

## 4. Discussion

In this study, we sought to explore expert clinicians’ views regarding reintegration interventions for CPTSD. The main aims related to conceptualising the definition, composition, and key principles of delivery of reintegration, due to limited previous research and inconsistencies on this topic. Experts’ definitions of reintegration included a range of ideas, but consistently discussed were themes relating to identity, transitioning skills to real life, being future-orientated, accessing resources, and, lastly, enhancing life. The composition of reintegration similarly consisted of a wide variety of suggestions. The agreement between all experts was that the interventions should be based on the individual, with only a few experts suggesting standardised interventions that may be used for everyone. Finally, the key principles of delivering reintegration were more consistent among the participants than the definition or composition.

Several themes, such as transferring skills from therapy to real life and enhancing the individual's life are consisted with Herman’s (1992) initial definition for reintegration, which discussed incorporating lessons learnt from therapy with the aim of helping the person move forward from their traumatic experiences while regaining a sense of control. This is also consistent with Cloitre et al.'s (2012) suggestion that reintegration relates to the transition out of therapy and into the community.

In terms of the existing literature, only limited studies have defined reintegration as developing identity and self-esteem (Courtois, 2010; Courtois & Ford, 2009). Expert clinicians in this study, however, consistently discussed the development of identity when defining reintegration, suggesting that this is an important part of reintegration which has been somewhat neglected in previous literature. Contrary to existing research, most experts also discussed the idea of reintegration being future-orientated. Some experts defined reintegration as the ‘moving forward’ phase, which has not been explicitly identified previously. This ultimately brings into question existing definitions of reintegration.

A consistent issue discussed related to whether to term this process either ‘integrating’ or ‘reintegrating’ back into the community. Many participants talked about ‘reintegration’ not being the correct word for the treatment, as it would assume that people had been previously integrated in life, which, according to the experts, often is not the case in CPTSD. This was consistently highlighted, with suggestions that ‘integration’ would be a more valid term for the treatment. Similarly, some experts stated that they used the terms ‘reclaiming’ or ‘rebuilding’ instead of ‘reintegration’. The rationale behind using these alternative terms was that during treatment the individual is more likely to participate during therapy with regards to building their future and ‘claiming’ the life that they want. The term ‘reintegration’ can suggest a more passive integration back into the community.

In terms of the composition of reintegration interventions, experts also varied greatly. Most participants agreed that interventions should be bespoke to the individual, with only one expert suggesting standardised interventions that may be used for everyone, such as interventions aimed at improving physical wellbeing and exercise. Contradicting most current suggestions for reintegration interventions found in the literature, consistent suggestions among experts related to occupation, functioning and social integration interventions. According to the recent systematic review by Purnell et al. (2021) which examined existing published research on reintegration interventions, the only previously researched interventions consisted of physical activity, including yoga, use of service dogs, residential treatment, education, self-defence, and patient involvement in research. The gap between what has been evaluated in research so far and what expert clinicians have suggested to use may go some way to explaining the inconsistent effectiveness of reintegration interventions.

There was clearer consensus among experts for certain key principles in delivering reintegration. This could suggest that regardless of the definition and composition of reintegration, experts follow the same set of principles in delivering reintegration interventions. According to most experts, reintegration should be delivered alongside other trauma-focused interventions, with the individual benefiting most when reintegration starts at the beginning of therapy, rather than it being delivered closer to the end of therapy. This view is consistent with the newly updated ISTSS (2019) guidelines, which do not explicitly recommend a phase-based approach, but instead adopt an integrative approach in treatment for CPTSD (Berliner et al., 2019).

A key principle in delivering reintegration among all experts was to take a person-centered approach which is tailored to the individual's experiences, needs and values. The ISTSS guidelines (2019) have suggested a ‘personalizing medicine’ approach to treating CPTSD, suggesting tailoring interventions to previously identified clinically significant symptoms of the individual (Berliner et al., 2019). Furthermore, the NICE guidelines (2018) on PTSD suggest that CPTSD treatment should consider the individual's safety and stability and should also build in extra time to develop trust depending on their needs and plan additional support after the end of treatment. These recommendations are consistent with the themes developed in our research.

Regarding the duration of the reintegration treatment, most experts expressed it being linked both to the person-centred approach and to the other phases of treatment. However, previous research has found that the most highly rated interval was three months, with the same study suggesting that reintegration should last between six and 12 months (Cloitre et al., 2011). Our results demonstrate that experts actually take a minimum of three months in most settings, with many suggesting that treatment may be significantly longer.

There is currently no existing literature regarding the evaluation of reintegration interventions, demonstrating the need to examine this question more thoroughly. As Purnell et al. (2021) have previously suggested, a measure for reintegration interventions could prove useful in comparing their effectiveness, it could also be beneficial in the evaluation of reintegration. All the expert clinicians expressed the value of reintegration in their practice along with need for more research on this topic.

### 4.1. Strengths and limitations

To our knowledge, this is the first study to explore expert opinion regarding reintegration interventions. Our study included 16 leading national and international clinical experts and academics in order to gather expert opinion regarding reintegration. Due to the lack of literature on this topic, our study has established new themes within the conceptualisation, composition and key principles of reintegration, that have not been explored previously. We also sought participants from a variety of settings, including different clinical roles, client groups with CPTSD and geographical locations with the aim of comparing similarities and differences in opinion between clinical settings which has not previously been examined. Due to the nature of the codebook thematic analysis used in the study, the results are detailed accounts of expert views on reintegration, which captured the consensus and disagreements between the participants. Finally, the codes were frequently discussed and refined between authors to maximise their validity.

Nonetheless, this study does have limitations that need to be considered. Our study only included 16 participants, due to the time constraints of the research. Out of the 16 participants, only four experts were based outside of the UK, limiting our ability to draw conclusions regarding agreement or disagreement in views based on clinical experience in different geographical locations.

### 4.2. Clinical implications

All experts consulted in this research agreed on the invaluable role reintegration plays within therapy for CPTSD. Whilst clear consensus on the definition and composition of reintegration has yet to be reached, the findings of this study lay the foundation for a framework of what reintegration is and how it can be used in the future.

Based on the experience and opinions of established international experts in CPTSD; reintegration interventions are essential for (re)building an individual's sense of self, transferring skills learned in therapy to everyday life, should be future-oriented and help the person to move forwards with their life, involve developing and accessing resources, and enhancing the person's life. The exact nature of reintegration interventions should be bespoke to the needs of the individual, but may include focus on improving functioning, social integration and building relationships, physical wellbeing and exercise, education and occupation. Goals for reintegration should be person-centered, achievable, meaningful and realistic, and should be collaboratively derived between the therapist and the individual, although may also involve others in their delivery. Reintegration should be considered from the beginning of therapy, although may be the more explicit focus of sessions towards the end of treatment.

We hope the study has provided insight for clinicians into the composition and key principles of delivery of reintegration interventions for use in their routine practice.

### 4.3. Research implications

It is important for future research to establish a consensus in defining reintegration along with its composition and preferred methods of delivery, which could be achieved by implementing a method such as a Delphi survey and using the results of the current study to reach an agreed consensus. Another recommendation for future research is to conduct qualitative research with clients with CPTSD to explore their views on reintegration, and integrate their opinions in further recommendations. Additionally, the lack of tools to evaluate reintegration suggests the need to investigate appropriate measures for evaluating reintegration interventions, which could be beneficial both in terms of clinical practice and in further research on reintegration.

### 4.4. Conclusion

This study provides an in-depth analysis of expert opinion regarding reintegration interventions, presenting a more developed view of reintegration, which has contradicted some of the previous research as well as yielding new insights in this field. The results of this study lay the foundation for a framework of what reintegration is and how it can be used, but also highlight the need for more research to be conducted on the role of reintegration in the treatment of CPTSD. Clear consensus on the definition and composition of reintegration is still yet to be reached. Possible measures for evaluating reintegration should also be explored in the future.

## Supplementary Material

Supplemental Material

## Data Availability

Data is available from JB upon reasonable request. Interview data has not been made generally available in order protect the anonymity of participants who are established international experts in CPTSD.
