# Supplementary material for: Expert international trauma clinicians’ views on the definition, composition and delivery of reintegration interventions for complex PTSD
Source: Eur J Psychotraumatol. 2023 Jan 17;14(1):2165024. doi: 10.1080/20008066.2023.2165024 (PMC9848321; doi:10.1080/20008066.2023.2165024)
Supplement: Supplemental Material [file ZEPT_A_2165024_SM4698.docx]

**Interview Schedule**

*Thank you for giving up your time to take part in this research. We are keen to hear your expert opinion as an experienced trauma clinician on phase three of the phase-based approach which previous experts have recommended for treatment of complex PTSD.*

*In existing guidance, experts have previously recommended a phase-based approach for working with people with complex PTSD, where the first phase is stabilisation, the second is trauma memory reprocessing, and the third reintegration. However, the third phase has received little attention or research to date. As an experienced clinician in treating CPTSD, we would like to explore your views about phase 3 reintegration interventions with the aim of coming up with a consensus of clinical experts on its definition, what it might include, and principles for its delivery.*

*This interview should take a maximum of 30 minutes. I will be audio recording the interview but will not retain any personally identifying information about you or your place of work in the transcript of the interview and you will not be identifiable from any publications or communications arising from this study.*

*Thank you and do you have any questions before we start?*

*Complete socio-demographic questions.*

1. How would you define phase 3 reintegration?

2. What, if anything, might reintegration work add to the treatment of CPTSD?

- In your opinion, does it enhance standard treatment for PTSD?

3. Have you used reintegration in your practice with clients with CPTSD?

- If so, can you give an example?

4. What sort of interventions would you consider to be a phase three reintegration intervention?

5. What do you think are the key principles in delivering phase three reintegration interventions?

- At what point in treatment should reintegration interventions be delivered?
- Who should deliver them?
- How long should they last?

6. How could/should phase 3 reintegration interventions be evaluated?

7. Is there anything else you would like to add?
